# Calcification in dermal fibroblasts from a patient with GGCX syndrome accompanied by upregulation of osteogenic molecules

**DOI:** 10.1371/journal.pone.0177375

**Published:** 2017-05-11

**Authors:** Yumi Okubo, Ritsuko Masuyama, Akira Iwanaga, Yuta Koike, Yutaka Kuwatsuka, Tomoo Ogi, Yosuke Yamamoto, Yuichiro Endo, Hiroshi Tamura, Atsushi Utani

**Affiliations:** 1 Department of Dermatology, Nagasaki University Graduate School of Biomedical Sciences, Nagasaki, Japan; 2 Research and Clinical Center for Yusho and Dioxin (ReC^2^YD), Kyushu University Hospital, Fukuoka, Japan; 3 Department of Molecular Bone Biology, Nagasaki University Graduate School of Biomedical Sciences, Nagasaki, Japan; 4 Department of Genetics, Research Institute of Environmental Medicine (RIeM), Nagoya University, Aichi, Japan; 5 Department of Healthcare Epidemiology Research, Graduate School of Medicine Kyoto University, Kyoto, Japan; 6 Department of Dermatology, Graduate School of Medicine Kyoto University, Kyoto, Japan; 7 Department of Ophthalmology and Visual Sciences, Graduate School of Medicine Kyoto University, Kyoto, Japan; University of California, Los Angeles, UNITED STATES

## Abstract

Gamma-glutamyl carboxylase (GGCX) gene mutation causes GGCX syndrome (OMIM: 137167), which is characterized by pseudoxanthoma elasticum (PXE)-like symptoms and coagulation impairment. Here, we present a 55-year-old male with a novel homozygous deletion mutation, c.2,221delT, p.S741LfsX100, in the *GGCX* gene. Histopathological examination revealed calcium deposits in elastic fibers and vessel walls, and collagen accumulation in the mid-dermis. Studies of dermal fibroblasts from the patient (GGCX dermal fibroblasts) demonstrated that the mutated GGCX protein was larger, but its expression level and intracellular distribution were indistinguishable from those of the wild-type GGCX protein. Immunostaining and an enzyme-linked immunosorbent assay showed an increase in undercarboxylated matrix gamma-carboxyglutamic acid protein (ucMGP), a representative substrate of GGCX and a potent calcification inhibitor, indicating that mutated GGCX was enzymatically inactive. Under osteogenic conditions, calcium deposition was exclusively observed in GGCX dermal fibroblasts. Furthermore, GGCX dermal fibroblast cultures contained 23- and 7.7-fold more alkaline phosphatase (ALP)-positive cells than normal dermal fibroblast cultures (n = 3), without and with osteogenic induction, respectively. Expression and activity of ALP were higher in GGCX dermal fibroblasts than in normal dermal fibroblasts upon osteogenic induction. mRNA levels of other osteogenic markers were also higher in GGCX dermal fibroblasts than in normal dermal fibroblasts, which including bone morphogenetic protein 6, runt-related transcription factor 2, and periostin (POSTN) without osteogenic induction; and osterix, collagen type I alpha 2, and POSTN with osteogenic induction. Together, these data indicate that GGCX dermal fibroblasts trans-differentiate into the osteogenic lineage. This study proposes another mechanism underlying aberrant calcification in patients with GGCX syndrome.

## Introduction

Gamma-glutamyl carboxylase (GGCX) activates a specific group of proteins (so-called gamma-carboxyglutamic acid (Gla) proteins) by adding a carboxyl group to the gamma position of glutamine [1]. Gla proteins include vitamin K-dependent coagulation factors (factors II, VII, IX, and X), protein C, protein S, and protein Z [2], as well as bone component proteins, such as matrix Gla protein (MGP) [3], osteocalcin (OCN) [4], periostin (POSTN) [5], and Gla-rich protein [6].

Loose, redundant skin and a bleeding tendency are observed in patients with GGCX syndrome. Vanakker et al. [7] reported six patients with a severe pseudoxanthoma elasticum (PXE)-like skin appearance, relatively mild angioid streaks, and perturbed vitamin K-dependent coagulation factor activities, which drew attention to the similarity between the skin and eye phenotypes in GGCX syndrome and PXE (OMIM: 264800) [8].

The clinical and pathological features that are similar in GGCX syndrome and PXE are also observed in other diseases, namely, generalized arterial calcification of infancy with *ectonucleotide pyrophosphatase/phosphodiesterase 1* mutation (OMIM: 208000) [9], calcification of joints and arteries caused by deficiency of CD73 (OMIM: 212800), fibrodysplasia ossificans progressiva due to activin A receptor type I deficiency (OMIM: 135100) [10, 11], haemoglobinopathy with *ß-globin* mutation (OMIM: 613985) [12], and congenital erythropoietic porphyria with *uroporphyrinogen III synthase* mutation (OMIM: 606938) [13].

The gamma position of certain glutamine residues of MGP is post-translationally carboxylated by GGCX [14]. Binding of γ-carboxylated (active) MGP to calcium ions and hydroxyapatite crystals inhibits calcium crystal formation [15, 16]. Deletion of the *MGP* gene in mice leads to spontaneous fragmentation and calcification of elastic fibers in the medial layer of arteries [17]. Therefore, undercarboxylated, inactive, MGP in GGCX syndrome is thought to be involved in ectopic calcification; however, the precise process of calcification of elastic fibers is not known.

The clinical and pathological features of GGCX syndrome are similar to those of PXE [8]. Although extensive studies have been performed, the mechanisms underlying calcification of elastic fibers in PXE have not been fully elucidated. In recent years, we have reported a relationship between cutaneous and mucous lesions and the incidence of cardiovascular symptoms in Japanese patients with PXE [18] as well as atypical cases of PXE [13, 19–21]. Through these experiences, we noticed that skin redundancy and calcification were more severe in the GGCX syndrome patient presented here than in PXE patients. There are no reports concerning calcium deposits using dermal fibroblasts from a patient with GGCX syndrome (GGCX dermal fibroblasts), although a few studies have been performed using dermal fibroblasts derived from patients with PXE (PXE dermal fibroblasts) [22, 23]. In the present study, we revealed enhanced *in vitro* calcification accompanied by upregulation of osteogenic marker expression [24–30] in GGCX dermal fibroblasts compared with normal dermal fibroblasts. This suggests that GGCX dermal fibroblasts trans-differentiate into osteogenic mesenchymal cells, which could be linked to ectopic calcification in the dermis.

## Materials and methods

The experiments in this study were performed with written informed consent, adherence to the Declaration of Helsinki protocols, and approval of the Ethical Committee of the Nagasaki University Graduate School of Biomedical Sciences.

### Picrosirius red staining

Paraffin sections were stained with a Picrosirius Red Stain Kit^®^ (Polysciences, Philadelphia, PA). Polarizing and light microscopy images were taken with a cellSens Standard platform (OLYMPUS, Tokyo, Japan).

### DNA sequencing

DNA was purified from blood samples using a Dneasy^®^ Blood & Tissue Kit (Qiagen, Hilden, Germany). Exon-specific primers for all 15 exons of *GGCX* were designed with primer3 (S1 Table). Sequencing reactions were performed with the following reaction conditions: 1 minute at 94°C; 30 cycles of 10 seconds at 95°C, 10 seconds at 55°C, and 1 minute at 72°C; and finally 5 minutes at 72°C. PCR products were bidirectionally sequenced with a Big-Dye^®^ Terminator v3.1 Cycle Sequencing Kit (Applied Biosystems, Foster City, CA) and an ABI PRISM^®^ 3130 Genetic Analyzer (Applied Biosystems).

### Cell culture

GGCX dermal fibroblasts were established by plating small pieces of excised skin from the abdomen of the GGCX patient on plastic dishes. Cells were grown in Dulbecco’s Modified Eagle’s Medium (DMEM; NISSUI PHARMACEUTICAL Co. Ltd., Tokyo, Japan) containing 10% fetal bovine serum (FBS; EQUITECH-BIO, Inc., Kerrville, TX), 1 mmol/L L-glutamine solution (Wako Pure Chemical Industries, Ltd. Osaka, Japan), 0.75% (w/v) sodium bicarbonate solution (Wako Pure Chemical Industries, Ltd.) and 100,000 U/L of penicillin and 100 mg/L of streptomycin (Penicillin-Streptomycin solution, Wako Pure Chemical Industries, Ltd.). The cultures were incubated at 37°C in a 5% CO_2_ incubator until cells grew out of the tissue pieces. These cells were maintained in the same culture medium, passaged by splitting at a ratio of 1:2–3 when they reached early confluency, and frozen in liquid nitrogen until use. Dermal fibroblasts from three healthy individuals (CON1-3; mean age, 62 years; male:female ratio, 2:1) were used to assess the number of alkaline phosphatase (ALP)-positive cells and for RT-PCR of ALP. Growth of CON3 slowed over time; therefore, dermal fibroblasts were obtained from another healthy individual (CON4) and used instead of CON3 (CON1, CON2, and CON4; mean age, 68 years; male:female ratio, 2:1) for ALP activity assays; RT-PCR analysis of MGP, bone morphogenetic protein (BMP) 2, BMP6 [31], runt-related transcription factor 2 (RUNX2) [26], osterix (OSX) [27], collagen type I alpha 2 (COL1A2) [28], POSTN, OCN, collagen type II alpha 1 (COL2A1) [32], collagen type X alpha 1 (COL10A1) [33], matrix metalloproteinase (MMP)-2, and MMP-9 [34] (S1 Fig); and the BMP inhibitor test. PXE dermal fibroblasts were also obtained from three PXE patients (S2 Table). All primary cultured cells were used at passage 3–8.

### Real-time PCR

Dermal fibroblasts (4.0 × 10^5^) were seeded on 6-well plates. When they reached early confluency, cells were cultured for 3 days in DMEM containing 10% FBS and 0.1 mM vitamin C (L-Ascorbic Acid Phosphate Magnesium Salt; Wako Pure Chemical Industries, Ltd.). For osteogenic induction, the medium was changed to induction medium as described below. After 4 days of induction, total RNA was extracted with an RNAeasy Kit (Qiagen) and cDNA was synthesized using Ready-To-Go RT-PCR Beads (GE Healthcare Bio-Sciences, Pittsburgh, PA). Expression levels of the following genes were quantitated by RT-PCR using TaqMan (R) Gene Expression Assays, Inventoried (Applied Biosystem): glyceraldehyde-3-phosphate dehydrogenase (GAPDH; Hs00266705_g1), GGCX (Hs00168139_m1), MGP (Hs00969490_m1), ALP liver/bone/kidney (Hs01029144_m1), BMP2 (Hs00154192_m1), BMP6 (Hs01099594_m1), RUNX2 (Hs01047973_m1), OSX (Hs01866874_s1), POSTN (Hs01566750_m1), bone Gla protein (OCN, Hs01587814_g1), COL2A1 (Hs00264051_m1), MMP2 (Hs01548727_m1), and MMP9 (Hs00234579_m1).

TaqMan probes and primers were mixed with TaqMan Universal PCR Master Mix (Applied Biosystems) according to the manufacturer’s instructions. For RT-PCR analysis of COL1A2 and COL10A1, RT-PCR products were analyzed using TaqMan probes (Roche Applied Science, Mannheim, Germany). The TaqMan probes and primers used were as follows: COL1A2: probe no. 54, forward primer (1,287–1,306 nt Genbank: J03464.1) 5’-TGGAGTCCGAGGACCTAATG-3’, and reverse primer (1,361–1,379 nt) 5’-CCGATATTTCCAGGGGAAC-3’; COL10A1: probe no. 6, forward primer (1–20 nt Genbank: NM_000493.3) 5’-CACCTTCTGCACTGCTCATC-3’, and reverse primer (84–104 nt) 5’GGCAGCATATTCTCAGATGGA-3’; and GAPDH: probe no. 60, forward primer (83–101 nt Genbank: NM_002046.3) 5’-AGCCACATCGCTCAGACAC-3’, and reverse primer (130–148 nt) 5’-GCCCAATACGACCAAATCC-3’. Triplicate reactions were performed in an AB7300 real-time thermocycler (Applied Biosystems) using the following reaction conditions: 1 minute at 94°C; 30 cycles of 10 seconds at 95°C, 10 seconds at 55°C, and 1 minute at 72°C; and finally 1 minute at 72°C.

### Western blotting

Dermal fibroblasts (4.0 × 10^5^) were seeded on 6-well plates. When they reached early confluency, cells were washed with cold phosphate-buffered saline (PBS) twice, lysed on ice for 5 minutes with RIPA buffer (#9806S, Cell Signaling Technology, Danvers, MA) containing protease inhibitors (Protease Inhibitor Cocktail Set I; Calbiochem, Darmstadt, Germany) and 1 mM phenylmethylsulfonyl fluoride (Santa Cruz, Dallas, TX), collected using a cell scraper, and vortexed for 30 seconds. The lysates were centrifuged at 12,500 rpm for 10 minutes at 4°C, and the supernatants were quantitated using a Quick Start Bradford protein assay (Quick Start BSA standard set, Bio-Rad Laboratories, Inc., Hercules, CA). Samples (8.0 μg of each) were mixed with SDS-sample buffer containing 2% β-mercaptoethanol and separated on 10% SDS-PAGE gels (BIO-RAD Mini-PROTEAN TGX Gels, Bio-Rad Laboratories, Inc.) after heat denaturation. Samples were not heated for analysis of GGCX because this protein could not be detected following heat denaturation. After electrophoresis, proteins were electrically transferred to PVDF membrane (Immobilon-P membrane, Merck Millipore Ltd., Darmstadt, Germany) with a Bio-Rad transfer system (Mini-PROTEAN II Cell, Bio-Rad Laboratories, Inc.). After blocking for 30 minutes in dilution buffer (PBS containing 5% non-fat dry milk and 0.1% Tween 20), membranes were incubated with an anti-GGCX antibody (1:500) (GTX109926; GeneTex, Inc., Irvine, CA) or an anti-β-actin antibody (1:2,000) (#4967; Cell Signaling Technology) diluted in dilution buffer for 1 hour, followed by incubation with peroxidase-conjugated anti-mouse or anti-rabbit secondary antibodies (1:2,000) (GE Healthcare Bio-Sciences) for 30 minutes. Signals were detected with ImageQuant LAS 4000 mini (GE Healthcare Bio-Sciences) following treatment with ECL Plus (#32132; Thermo Fisher Scientific Inc., Waltham, MA).

### Immunocytostaining

Cells on coverglasses were treated with 4% paraformaldehyde (Nacalai Tesque, Inc., Kyoto, Japan) for 10 minutes at room temperature and permeabilized with 0.5% Triton X-100 (Nacalai Tesque, Inc.) for 7 minutes at room temperature. For staining with the anti-ucMGP antibody, cells were fixed in acetone at -20°C for 5 minutes. After washing with PBS three times, cells were treated with blocking buffer (PBS containing 10% FBS) for 30 minutes at room temperature, followed by incubation with the primary antibody diluted in blocking buffer overnight at 4°C or for 1 hour at room temperature. The primary antibodies were diluted 1:50–100, including anti-GGCX (GTX109926; GeneTex, Inc.), anti-58K Golgi (ab27043; Abcam, Cambridge, UK), anti-MGP (sc-271906; Santa Cruz), and anti-ucMGP (#ALX-804-642, mAb (BD4.4); Enzo Life Sciences, Antwerp, Belgium) [35] antibodies. Samples were reacted with secondary antibodies (1:200) (goat anti-mouse or rabbit IgG (H+L), Alexa Fluor^®^ 488- or 546-conjugated; Thermo Fisher Scientific Inc.) for 30 minutes at room temperature with or without Hoechst (H3570, Life Technologies Japan Ltd, Tokyo, Japan). After washing, samples were mounted in anti-fade solution (fluorescence mounting medium; Dako, Glostrup, Denmark) and images were taken with an OLYMPUS BX50 microscope (OLYMPUS), or a Keyence BZ-9000 Fluorescence Microscope (Keyence, Osaka, Japan).

### Enzyme-linked immunosorbent assay (ELISA) for ucMGP

ucMGP concentrations in GGCX dermal fibroblasts were quantified using a human ucMGP ELISA Kit according to the manufacturer’s protocol (#CSB-EC013789HU, Cusabio Biotech Co., Wuhan, China). Briefly, cells (6.4 × 10^5^) isolated from three controls and one GGCX patient were separately seeded on 6-well plates. When they reached early confluency, cells were washed with cold PBS twice and lysed on ice for 5 minutes with RIPA buffer (#9806S, Cell Signaling Technology) containing protease inhibitors (Protease Inhibitor Cocktail Set I; Calbiochem) and 1 mM phenylmethylsulfonyl fluoride (Santa Cruz). The lysate were collected using a cell scraper and vortexed for 30 seconds. After centrifugation at 12,500 rpm for 10 minutes at 4°C, protein concentrations in the supernatants were quantitated using a Quick Start Bradford protein assay (Quick Start BSA standard set, Bio-Rad Laboratories, Inc.). Samples (approximately 40 μg of protein) were mixed with the sample diluent, and the ucMGP content was assessed. Reactions were stopped by addition of acidic stop solution, followed by measurement of OD at 450 nm in triplicate (Labsystems Multiskan MS #352, Thermo Fisher Scientific Inc.). The values were adjusted by the protein contents, which were determined from the same plates.

### *In vitro* calcification assay

For mineralization experiments, cells were seeded at a density of 1.6 × 10^5^ cells/well in a 4-well chamber (Lab-Tek^®^ II Chamber Slide System 154526; Nunc, Rochester, NY). Cells were cultured for 7 days in DMEM containing 10% FBS and 0.1 mM vitamin C (L-Ascorbic Acid Phosphate Magnesium Salt; Wako Pure Chemical Industries, Ltd.). Vitamin C was added to obtain multilayered fully confluent fibroblasts. For induction, the medium was changed to osteogenic induction medium (10% FBS-supplemented DMEM containing 10 mM ß-glycerophosphate (Sigma-Aldrich, St. Louis, MO), 0.1 mM vitamin C (Wako Pure Chemical Industries, Ltd.), 10^−8^ M dexamethasone (Sigma-Aldrich), and 1% glutamine (Wako Pure Chemical Industries, Ltd.)). At 0, 7, and 14 days after induction, phase contrast images of cells, as well as those of von Kossa staining at 11 days, were taken with a Leica MC120HD camera (Leica microsystem, Tokyo, Japan).

### Detection of ALP-positive cells

One milliliter containing 1.6 × 10^5^ human cells and 1.0 × 10^5^ periosteoblasts was plated per well in a 24-well plate, and cells were allowed to grow for 3 days in DMEM containing 10% FBS and 0.1 mM vitamin C. Thereafter, the medium was replaced with osteogenic induction medium. At 4 days after induction, cells were fixed with 4% paraformaldehyde prepared in PBS for 10 minutes at room temperature. Thereafter, ALP activity was detected by incubating samples at room temperature for up to 20 minutes before background labeling appeared with ALP staining solution containing 0.1 mg/ml Naphthol AS-MX phosphate (Sigma-Aldrich), 0.5% N,N-dimethyl formamide (Nacalai Tesque, Inc.), 0.2 M Tris-HCl (pH 8.5) (Nacalai Tesque, Inc.), and 1.2 mg/ml Fast Blue BB (Sigma-Aldrich). Images were taken with a Leica MC120HD camera. ALP activity was quantified by counting the number of ALP-positive cells in ten randomly selected high-power fields. As a positive control, primary periosteoblasts from neonatal C57BL/6 mouse calvarias [36] were used in parallel experiments.

### ALP activity assay

ALP activity was assayed using LabAssay™ ALP according to the manufacturer’s protocol (#291–58601, Wako Pure Chemical Industries, Ltd.). Briefly, three control, three PXE, and one GGCX fibroblast cell lines were seeded in triplicate (1.6 × 10^5^ per well in a 48-well plate) and underwent osteogenic induction for 3 days starting the next day. Thereafter, cells were lysed by two freeze-thaw cycles with 150 μl of 0.05% Triton X-100 prepared in buffer (2.0 mmol/L MgCl2 and 0.1 mol/L carbonate buffer, pH 9.8). After centrifugation at 15,000 rpm for 15 minutes, supernatants (20 μl) were incubated for 25 minutes with ALP substrate (6.7 mmol/L p-nitrophenylphosphate disodium). Reactions were stopped by addition of stop solution (0.2 mol/L sodium hydroxide solution), followed by measurement of OD at 405 nm in triplicate (Labsystems Multiskan MS #352, Thermo Fisher Scientific Inc.). The values were adjusted by cell numbers, which were obtained by analysis of cells seeded in parallel.

### MTS assay

The optimum dorsomorphin concentration was determined using the CellTiter 96^®^ AQ_ueous_ One Solution Cell Proliferation Assay (G3580, Promega, Madison, WI) containing a tetrazolium compound (MTS). According to the manufacturer’s protocol, fibroblasts were seeded at a density of 1 × 10^4^ cells per well in a 96-well plate with DMEM and treated with dorsomorphin (0, 0.1, 1.0, 1.5, 2.0, 2.5, 3.0, 5.0, 10, 50, and 100 μM) for 24 and 48 hours. Thereafter, 20 μl of MTS reagent (317 μg/ml) was added to each well and incubated at 37°C for 4 hours in a humidified atmosphere containing 5% CO_2_. After adding 25 μl of stop solution (10% SDS-HCl), absorbance at 490 nm was recorded using an enzyme-linked immunosorbent assay (ELISA) plate reader.

### Statistical analyses

The results are given as means ± standard deviation (SD). Statistical analyses were performed using the unpaired, two-tailed student’s *t* test. *P* < 0.05 was considered statistically significant.

## Results

### Case report

**Fig 1:**
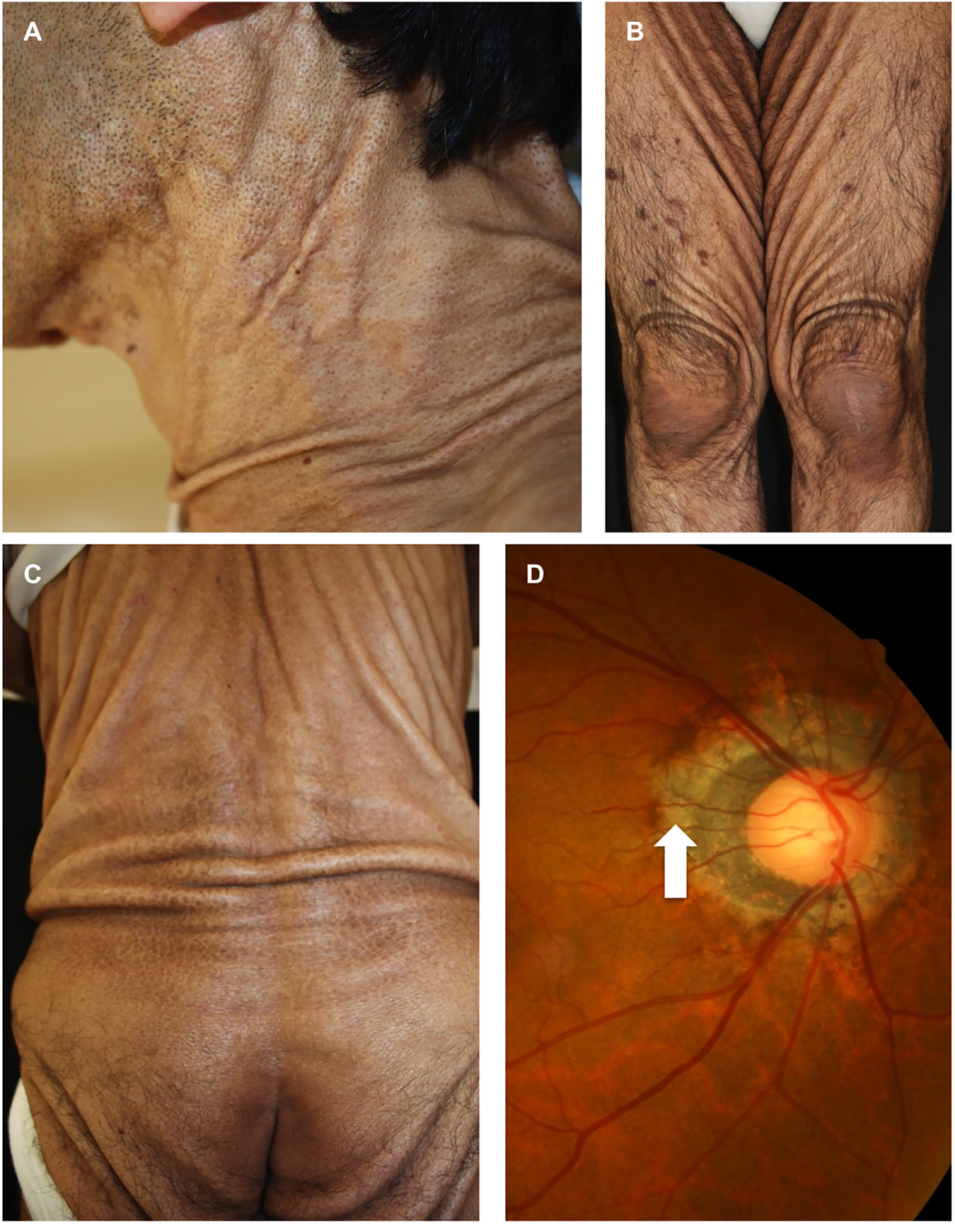
Clinical features and fundoscopy. Loose and sagging skin around the neck (A), legs (B), and back and hips (C). Peripapillary atrophy of the retina (arrow, D).

A 55-year-old Japanese male visited the Department of Neurology because of dysarthria and motor ataxia of the eye, trunk, and extremities, which had gradually worsened over the previous 4 years. His parents were cousins. A computed tomography scan of the chest and magnetic resonance imaging angiography of the brain demonstrated no abnormalities. ^123^I IMP-SPECT did not find low circulation in the cerebella, but stereotactic surface projections demonstrated slightly insufficient circulation in the frontal and temporal lobes. He was temporarily diagnosed with a spinocerebellar degeneration disorder to explain the motor symptoms, except for the eye movement disturbance. Physical examination found loose, sagging skin, like that observed in cutis laxa, over almost his entire body (Fig 1A–1C). Fundoscopy revealed peripapillary atrophy of the retina (Fig 1D) and no changes such as angioid streaks and peau d’orange, which are seen in PXE.

Laboratory data found coagulation abnormalities with a prothrombin time of 27.7 seconds (normal, 10–11 seconds), a prothrombin time-international normalized ratio of 4.07 (normal, 1.0), and an activated partial thromboplastin time of 49.7 seconds (normal, 30–40 seconds); low vitamin K-associated coagulation factors activities with factor II, 17%; factor VII, 6%; factor IX, 23%; and factor X, 12%; and urine abnormalities with protein, 2+; sugar, 2+; and occult blood, 2+. However, renal function data were within normal ranges, such as 0.8 mg/dl creatinine (normal, 0.6–1.2 mg/dl), 4.1 mg/dl uric acid (normal, 3.0–7.2 mg/dl), 17 mg/dl blood urea nitrogen (normal, 9–21 mg/dl), and an estimated glomerular filtration rate of 78.8. Neurological examinations showed abnormal extraocular movements, with severe restriction of supraduction and abduction, and slight restriction of adduction. There was Babinski reflex (+/+) in both feet and tandem gait failure. There is no report of a patient with GGCX syndrome who had neurological symptoms or abnormal urinalysis findings. Therefore, these symptoms might be caused by other unknown gene mutations because his parents were cousins.

### Histopathological studies of cutaneous lesions

**Fig 2:**
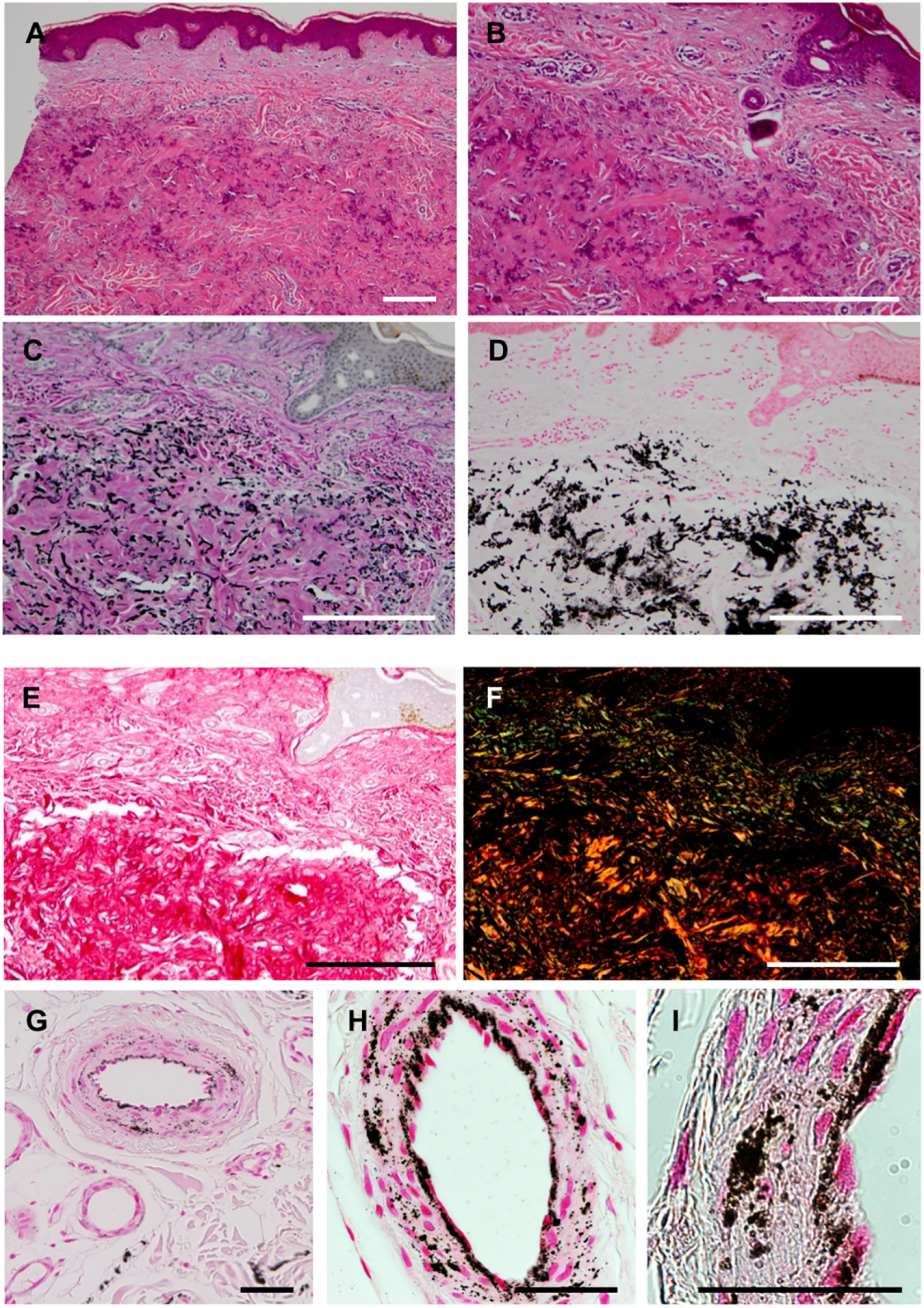
Histopathology of skin lesions. A biopsied specimen was stained with hematoxylin-eosin (A, B), Elastica van Gieson (C), von Kossa (D, G–I), and picrosirius red (E, F). The swollen collagen bundles were colored yellow−orange in polarizing microscopy (F). In the deeper dermis, the internal elastic lamina and a part of the arteriole walls were positive for calcification (G–I). The bar depicts 500 μm in (A–F) and 50 μm in (G–I).

A skin biopsy taken from the left side of the abdomen demonstrated basophilic amorphous materials intermingling with eosinophilic fibrous components in the mid-dermis (Fig 2A and 2B). Degenerative elastic fibers were observed by Elastica van Gieson staining (Fig 2C), and massive calcium deposits as well as elastic fiber calcification were visualized by von Kossa staining (Fig 2D). An increased number of swollen collagen bundles was also observed by picrosirius red staining (Fig 2E). The swollen collagen bundles were composed of thick fibers, as evidenced by the yellow−orange color in polarizing microscopy images of picrosirius red staining (Fig 2F). In the deeper dermis, the internal elastic lamina and a part of the arteriole walls, but not the sweat glands, were positive for calcification (Fig 2G−2I). These changes were not accompanied by the upregulation of MMP-2 or MMP-9 (S1 Fig).

### Detection of a homozygous deletion mutation in *GGCX* by DNA analysis

**Fig 3:**
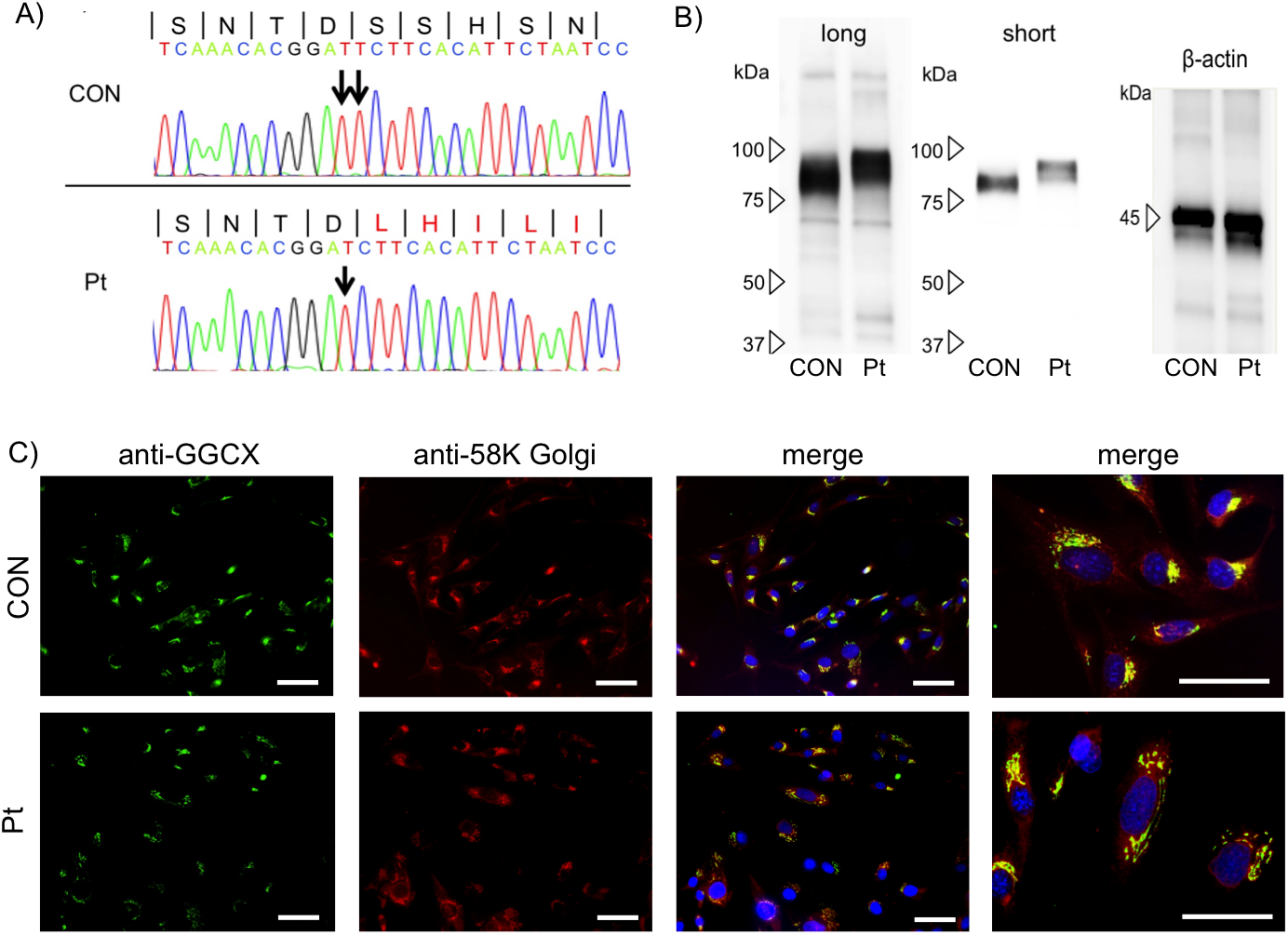
Characterization of the mutated GGCX protein. (A) A homozygous deletion of T in the *GGCX* gene in the patient (Pt): c.2,221delT, p.S741Lfsx100. The wild-type DNA sequence is also shown (CON), and the peptide sequence is depicted at the top. (B) Western blotting. Long: long exposure; short: short exposure. (C) Immunofluorescence staining. The mutated and wild-type GGCX proteins (anti-GGCX) were identically localized at the Golgi apparatus (anti-58K Golgi). The bar depicts 50 μm (original magnification, ×40).

Based on the clinical symptoms, laboratory data, and pathological findings, we suspected he had GGCX syndrome. Thus, we performed genomic sequencing of the *GGCX* gene and revealed a homozygous deletion of T in exon 15: c.2,221delT, p.S741LfsX100 (Fig 3A). This deletion alters the amino acid sequence from 741 to 758 amino acids. This mutation site is in the endoplasmic reticulum (ER) lumen of GGCX localized at the ER membrane [37] This novel mutation was expected to add 100 abnormal amino acids to the C-terminus (S2 Fig). Gene analyses of his parents were not available.

### Expression and intracellular localization of mutated GGCX

The mRNA level of mutated GGCX protein was unchanged in GGCX dermal fibroblasts compared with normal dermal fibroblast (S3 Fig) Consistently, western blotting demonstrated that the mutated GGCX protein was stably expressed and had a larger molecular weight than the wild-type GGCX protein (Fig 3B). In immunofluorescence analysis, mutated GGCX was localized at the Golgi apparatus, as evidenced by immunostaining with an anti-58K Golgi marker antibody, which was identical to the localization of wild-type GGCX in normal dermal fibroblasts (Fig 3C). Although GGCX was reported to be a membrane protein of the ER [38], our results indicated that mutated as well as wild-type GGCX in dermal fibroblasts were predominantly located at the Golgi apparatus membrane. Based on these observations, although the homozygous c.2,221delT mutation drastically changed the structure of the C-terminus of GGCX, it did not seriously affect its protein level or intracellular distribution. We next analyzed the functions of the mutated GGCX protein.

### An increased level of ucMGP indicates that the function of the mutated GGCX protein is impaired

**Fig 4:**
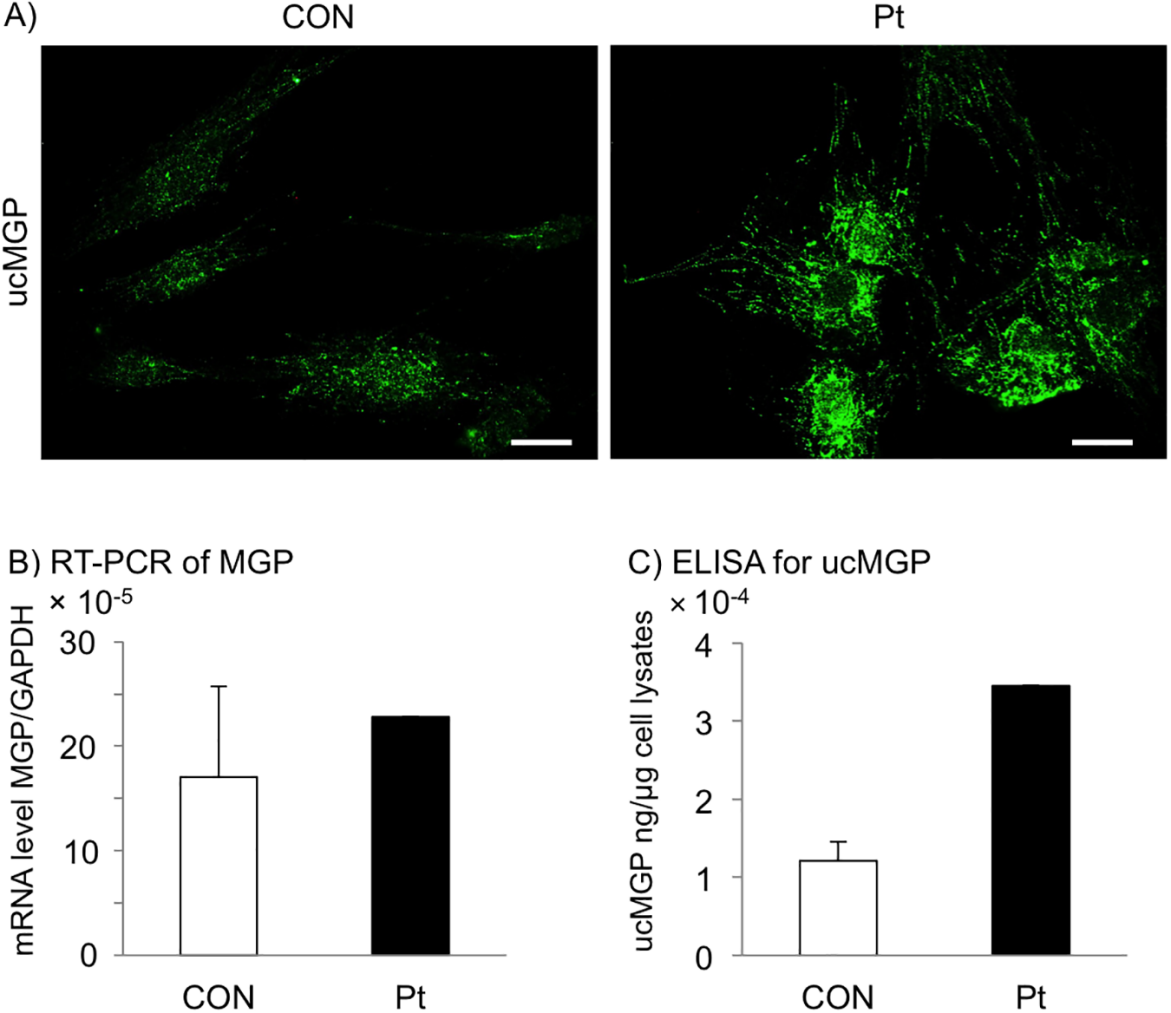
Increased level of ucMGP in GGCX dermal fibroblasts. (A) Immunofluorescence staining with an antibody specific for ucMGP. The level of ucMGP was higher in GGCX dermal fibroblasts (Pt) than in normal dermal fibroblasts (CON). The bar depicts 50 μm (original magnification, ×400). (B) RT-PCR analysis of MGP mRNA levels in normal (CON) and GGCX (Pt) dermal fibroblasts. The MGP mRNA levels in normal dermal fibroblasts are shown as mean ± SD (n = 4) and those in GGCX dermal fibroblasts as mean. (C) Quantification of ucMGP in normal (CON) and GGCX (Pt) dermal fibroblasts. Values in normal dermal fibroblasts are shown as mean ± SD (n = 3) and those in GGCX dermal fibroblasts as mean. These experiments were performed at least twice, and a representative data set is shown.

MGP is an anti-calcification factor and one of the most well-characterized substrates of GGCX, and the gamma-glutamyl residues of MGP are uncarboxylated in GGCX syndrome [39, 40]. Therefore, we attempted to show an increase in ucMGP in GGCX dermal fibroblasts, which would reflect dysfunction of the mutated GGCX protein. Immunofluorescence staining of ucMGP showed strong signals in GGCX dermal fibroblasts, but only faint signals in normal dermal fibroblasts (Fig 4A).

The MGP mRNA level in normal dermal fibroblasts was 17 × 10^−5^ ± 8.6 × 10^−5^, and the level in GGCX dermal fibroblasts was 23 × 10^−5^, which was within the mean ± 1SD of that in normal dermal fibroblasts (Fig 4B). By contrast, the ucMGP concentration in normal dermal fibroblasts was 1.2 × 10^−4^ ± 0.2 × 10^−4^ ng/μg cell lysate, and that in GGCX dermal fibroblasts was 3.5 × 10^−4^ ng/μg cell lysate, which was higher than the mean + 2SD of that in normal dermal fibroblasts (Fig 4C). Taking these results together, the significant increase in ucMGP did not merely reflect a relative increase of MGP, but indicated that GGCX function was severely abrogated in GGCX dermal fibroblasts.

### Accelerated calcification and upregulated ALP in GGCX dermal fibroblasts

**Fig 5:**
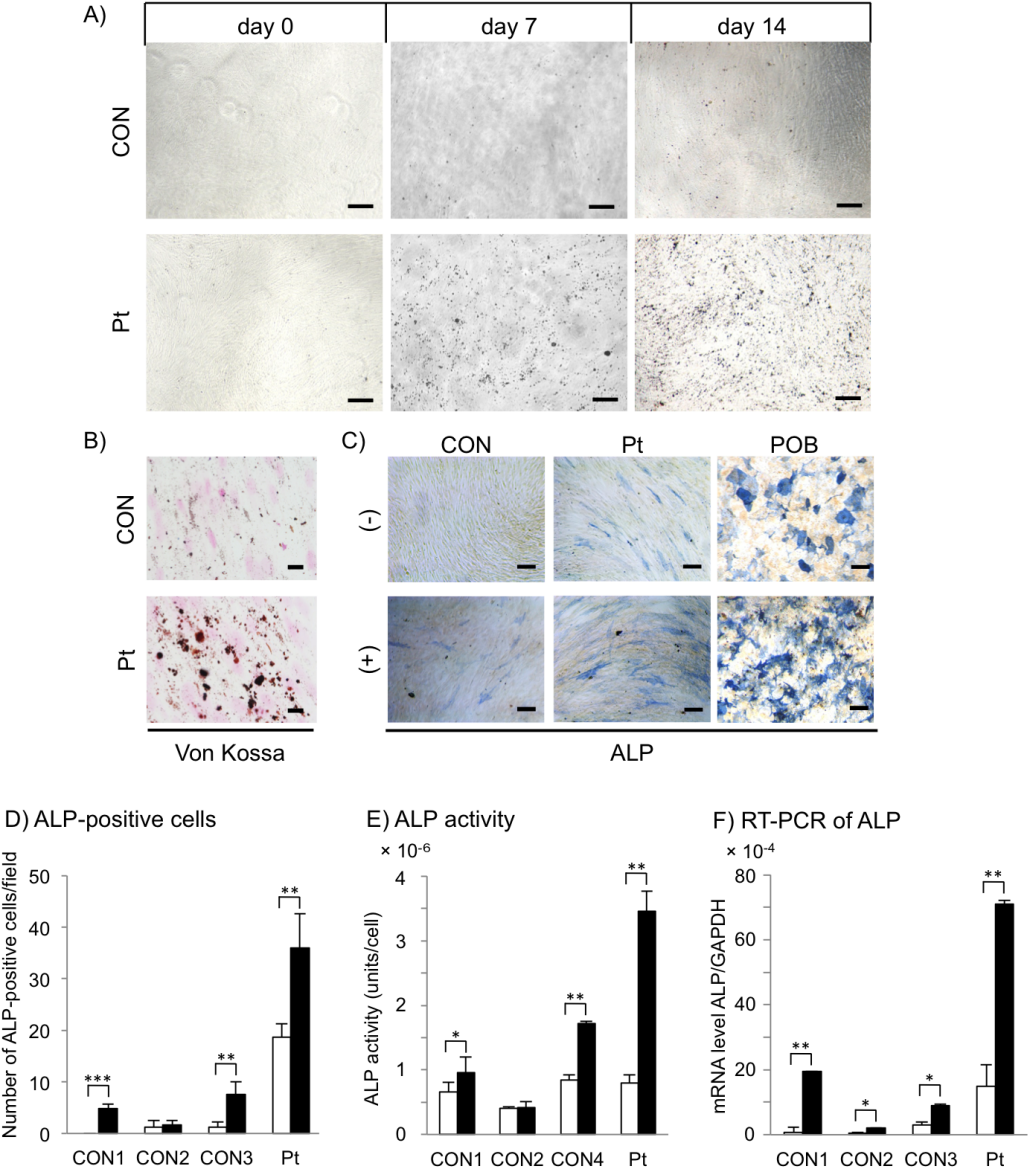
Accelerated calcification and upregulation of ALP in GGCX dermal fibroblasts. (A) There were more mineral particles in GGCX dermal fibroblasts (Pt) than in normal dermal fibroblasts (CON) after 7 and 14 days of osteogenic induction. (B) Particles were positive for von Kossa staining at 11 days after induction. (C) ALP staining of normal and GGCX dermal fibroblasts at day 4 with (+) or without (-) induction. Periosteoblasts (POB) were positive controls for osteogenic induction. The bar depicts 100 μm in (A, C) (original magnification, ×40) and 20 μm in (B) (original magnification, ×100). (D) ALP-positive cells were counted in ten randomly selected fields. 1 field = 12.56 mm^2^. (E) ALP activity analysis. Assays were performed using three different normal dermal fibroblast cell lines (CON1, CON2, and CON4) at day 4 without (open columns) or with (closed columns) osteogenic induction. Results are shown as mean ± SD. (F) Real-time PCR analysis of ALP. Assays were performed using three different normal dermal fibroblast cell lines (CON1–3) at day 4 without (open columns) or with (closed columns) osteogenic induction. Results are shown as mean ± SD. *: *P* < 0.05, **: *P* < 0.005, and ***: *P* < 0.0005. These experiments were performed at least twice, and a representative data set is shown.

*In vitro* calcification is reported to be abruptly enhanced with osteogenic induction in PXE dermal fibroblasts [23]; therefore, we performed *in vitro* calcification assays using GGCX dermal fibroblasts. Osteogenic commitment of cultured dermal fibroblasts was verified by time-serial phase contrast images, indicating that precipitates had already appeared at day 7 after osteogenic induction of GGCX dermal fibroblasts (Fig 5A). These changes further increased until day 14 (Fig 5A). Von Kossa staining verified that precipitates consisted of calcium deposits at day 11 in GGCX dermal fibroblasts, but not in normal dermal fibroblasts (Fig 5B). Next, the osteogenic properties of cultured dermal fibroblasts were examined by counting ALP-positive cells at day 4 (Fig 5C). ALP is expressed prior to calcium deposition in bone and calcifying cartilage [41]. For example, an *in vitro* study [42] reported that ALP activity in mesenchymal stem cells peaks at 3 days and declines at 7 days after osteogenic induction. Moreover, it was reported that calcium deposition is highest at 14 days after osteogenic induction, but no deposition is observed at 7 days [42]. Based on these observations, we decided to investigate ALP expression and activity at 4 days after induction, not at 7 or 14 days as we observed calcium deposition (Fig 5). Without osteogenic induction, the number of ALP-positive cells per field was 19 ± 2.6 among GGCX dermal fibroblasts, 0 in CON1, 1.2 ± 1.3 in CON2, and 1.2 ± 1.1 in CON3 (Fig 5D, open columns). The number of ALP-positive cells among GGCX dermal fibroblasts was 23-fold higher than the average number in the three normal dermal fibroblast samples. At day 4 of osteogenic induction, the number of ALP-positive cells increased among both cell types; there were 36 ± 6.7 ALP-positive cells per field among GGCX dermal fibroblasts, 4.8 ± 0.84 in CON1, 1.6 ± 0.89 in CON2, and 7.6 ± 2.4 in CON3 (Fig 5D, closed columns). The number of ALP-positive cells among GGCX dermal fibroblasts was 7.7-fold higher than the average number in the three normal dermal fibroblast cell lines.

Without osteogenic induction, the level of ALP activity in GGCX dermal fibroblasts was similar to that in normal dermal fibroblasts (Fig 5E, open columns). With osteogenic induction, it was 3.5 × 10^−6^ ± 0.31 × 10^−6^ unit/cell in GGCX dermal fibroblasts (Fig 5E, closed columns). ALP activity in GGCX dermal fibroblasts was 1.3-fold and 3.4-fold higher than the average level in normal dermal fibroblasts without and with osteogenic induction, respectively.

In RT-PCR analysis without and with osteogenic induction, the ALP mRNA level in GGCX dermal fibroblasts was 11-fold and 7.0-fold higher than the average level in normal dermal fibroblasts, respectively (Fig 5F).

### Expression of osteogenic trans-differentiation markers in GGCX dermal fibroblasts

To investigate these observations further, we analyzed the expression levels of other markers of osteogenic and chondrogenic differentiation, including BMP6, RUNX2, OSX, COL1A2, POSTN, OCN, BMP2, COL2A1, and COL10A1. BMPs are potent osteogenic factors [30], and it was reported that BMP6 is drastically upregulated in dermal fibroblasts, but not osteoblasts, after 3 days under conditions for vitamin D3-induced trans-differentiation into osteoblasts and that ALP is upregulated in a BMP6-dependent manner [25].

**Fig 6:**
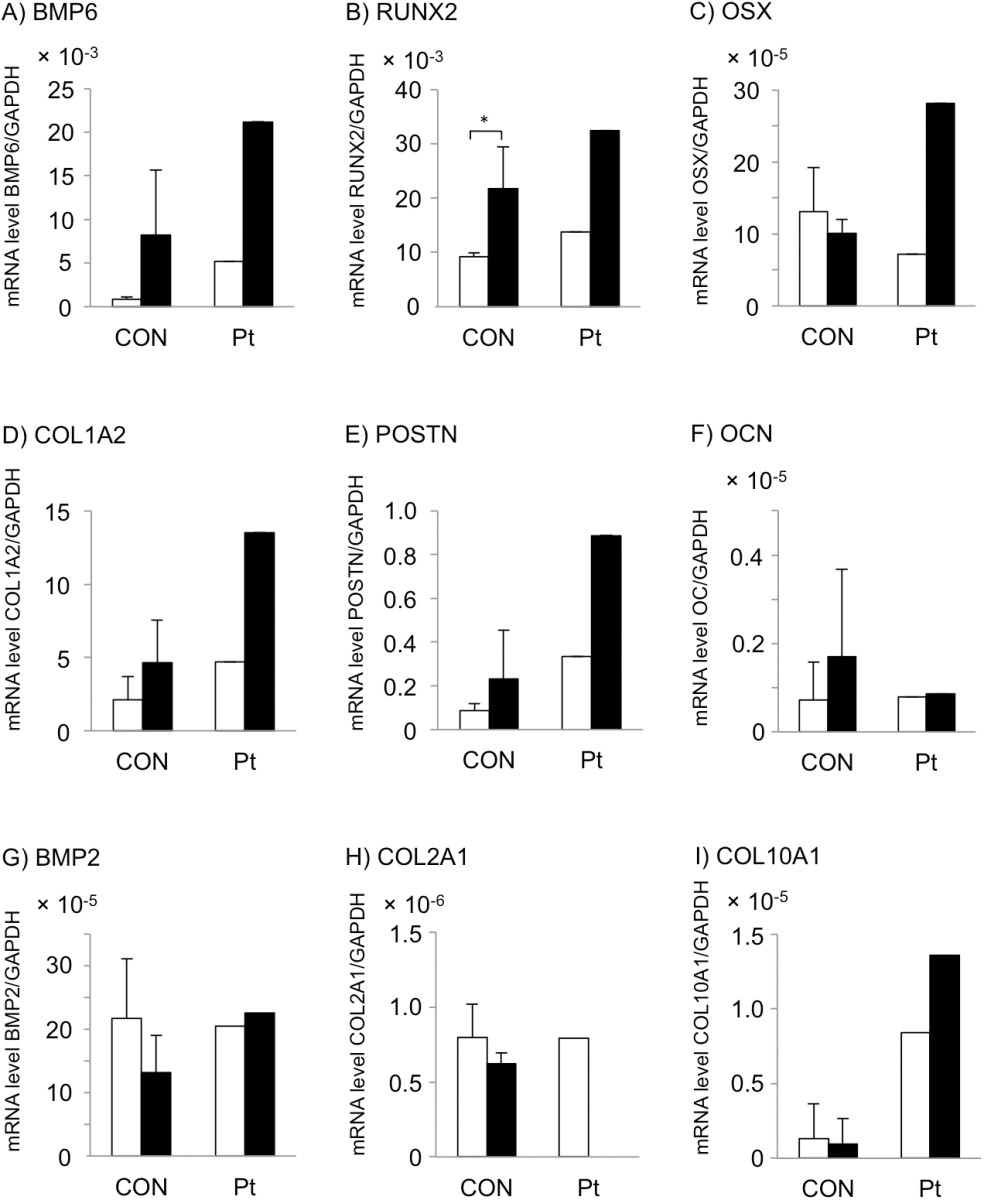
Expression of osteogenic trans-differentiation markers in GGCX dermal fibroblasts. RT-PCR analysis of BMP6 (A), RUNX2 (B), OSX (C), COL1A2 (D), POSTN (E), OCN (F), BMP2 (G), COL2A1 (H), and COL10A1 (I) in normal dermal fibroblasts (CON, n = 3) and GGCX dermal fibroblasts (Pt) at day 4 without (open columns) or with (closed columns) osteogenic induction. Values are mean ± SD for normal dermal fibroblasts and mean for GGCX dermal fibroblasts. *: *P* < 0.05. These experiments were performed at least twice, and a representative data set is shown.

Without osteogenic induction, expression levels of the osteogenic markers BMP6, RUNX2, and POSTN, and the late stage chondrogenic marker, COL10A1, were higher in GGCX dermal fibroblasts than the mean + 2SD of those in normal dermal fibroblasts (Fig 6A, 6B, 6E and 6I, open columns). The other five markers (OSX, COL1A2, OCN, BMP2, and COL2A1) were not upregulated in GGCX dermal fibroblasts without osteogenic induction. After osteogenic induction, mRNA expression levels of BMP6 and RUNX2 were higher in GGCX dermal fibroblasts than in normal dermal fibroblasts, but the difference was less than the mean + 2SD (Fig 6A and 6B, closed columns). mRNA expression levels of OSX, COL1A2, POSTN, and COL10A1 were higher in GGCX dermal fibroblasts than the mean + 2SD of those in normal dermal fibroblasts (Fig 6C–6E and 6I, closed columns). OCN and COL2A1 were expressed very weakly and were not upregulated even with osteogenic induction in either cell type (Fig 6F and 6H).

RUNX2 was significantly upregulated in normal dermal fibroblasts with osteogenic induction (Fig 6, CON). On the other hand, expression levels of, all molecules, except OCN, BMP2, and COL2A1, were increased by 1.6−4.1-fold in GGCX dermal fibroblasts upon osteogenic induction (Fig 6, Pt).

Many markers of osteogenic differentiation were upregulated in GGCX dermal fibroblasts regardless of osteogenic induction. This suggests that they differentiated into osteogenic mesenchymal cells, but not into osteocytes because they did not express OCN.

### ALP expression and activity are higher in GGCX dermal fibroblasts than in PXE dermal fibroblasts

**Fig 7:**
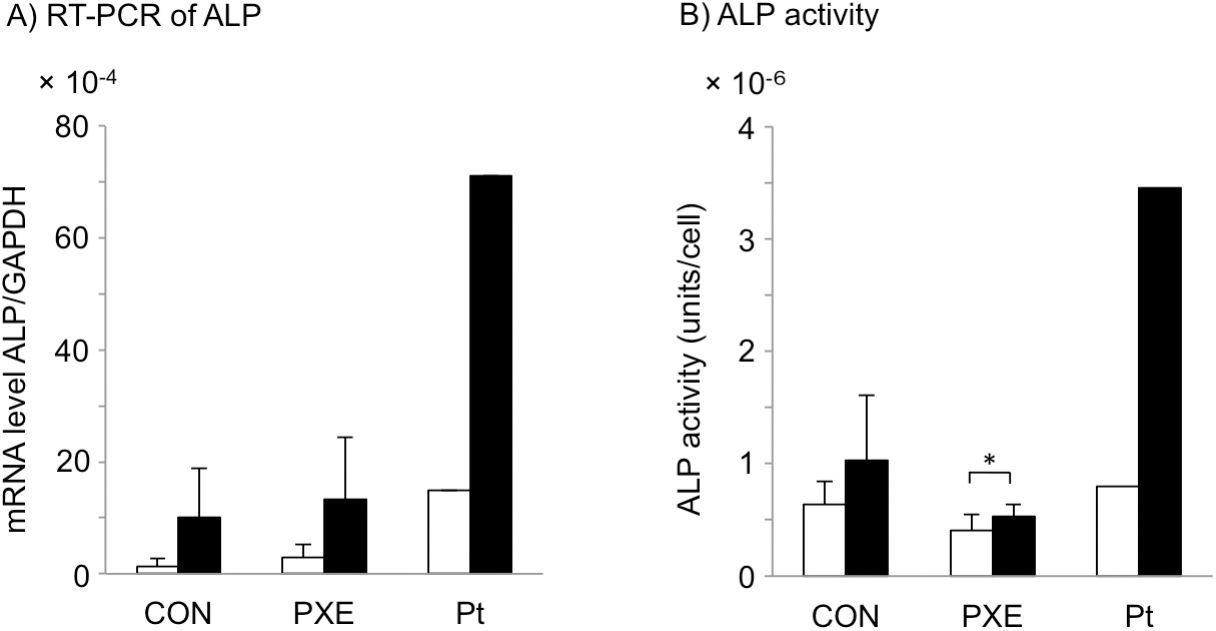
ALP expression and activity are higher in GGCX dermal fibroblasts than in PXE dermal fibroblasts. RT-PCR analysis of ALP mRNA levels (A) and analysis of ALP activities (B) without (open columns) or with (closed columns) osteogenic induction for 4 days in normal (CON, n = 3), PXE (PXE, n = 3), and GGCX (Pt) dermal fibroblasts. Values are mean ± SD for normal and PXE dermal fibroblasts and mean for GGCX dermal fibroblasts. The ALP mRNA expression levels in GGCX dermal fibroblasts were transferred from Fig 5F for comparison. *: *P* < 0.05. These experiments were performed at least twice, and a representative data set is shown.

Recently, it was shown that ALP activity is higher in PXE dermal fibroblasts than in normal dermal fibroblasts [22, 23]. We compared the mRNA expression and activity of ALP between GGCX and PXE dermal fibroblasts (S2 Table) after 4 days with or without osteogenic induction (Fig 7A and 7B). Without osteogenic induction, the average ALP mRNA level was higher in PXE dermal fibroblasts than in normal dermal fibroblasts, but the difference was not significant (*P* = 0.56). As described earlier, the ALP mRNA level in GGCX dermal fibroblasts was higher than the mean + 2SD of that in PXE dermal fibroblasts (Figs 5F and 7A, open columns). With osteogenic induction, the average ALP mRNA levels in control and PXE dermal fibroblasts were similar (Fig 7A, closed columns), while the ALP mRNA level in GGCX dermal fibroblasts was higher than the mean + 2SD of that in PXE dermal fibroblasts (Figs 5F and 7A, closed columns).

After osteogenic induction, ALP activity was remarkably upregulated in GGCX dermal fibroblasts, whereas changes in ALP activity were less pronounced in both CON and PXE dermal fibroblasts (Fig 7B, closed columns). Average ALP activity in control and PXE dermal fibroblasts was 1.0 × 10^−6^ ± 0.58 × 10^−6^ and 0.52 × 10^−6^ ± 0.11 × 10^−6^ units/cell, respectively (Fig 7B, closed columns), whereas it was 3.5 × 10^−6^ units/cell in GGCX dermal fibroblasts (Figs 5E and 7B, closed columns), which was higher than the mean + 2SD of that in PXE dermal fibroblasts.

These *in vitro* observations suggested that GGCX dermal fibroblasts have a stronger tendency than PXE dermal fibroblasts to trans-differentiate into osteogenic mesenchymal cells.

### Inhibition of BMP signaling blocks accelerated calcification

**Fig 8:**
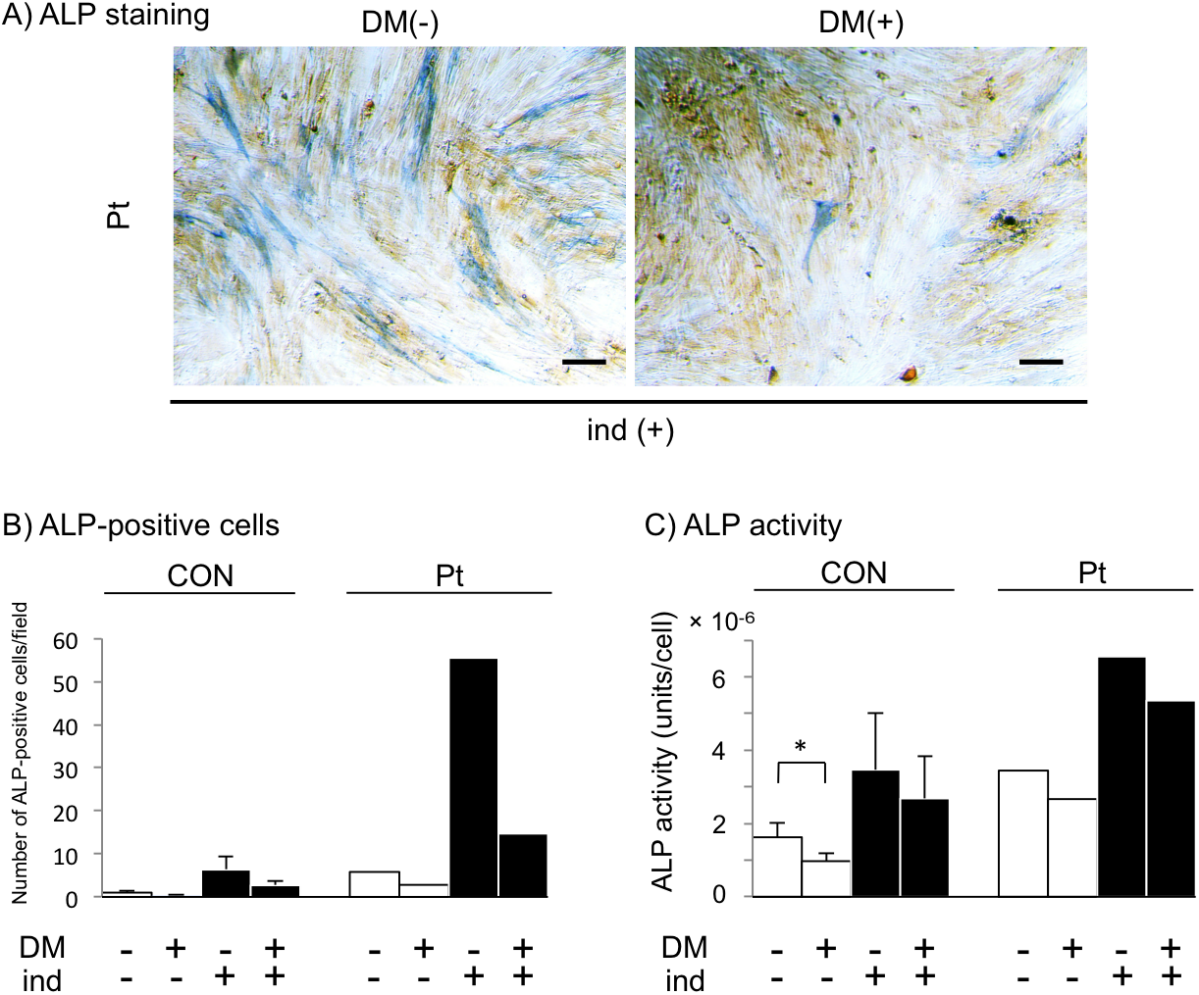
Inhibition of BMP signaling blocks accelerated calcification. ALP staining of GGCX (Pt) dermal fibroblasts (A), ALP-positive cells (B), and ALP activity (C) of both normal (CON, n = 3) and GGCX (Pt) dermal fibroblasts treated with 2 μM dorsomorphin (DM(+)) for 4 days were reduced regardless of induction (ind (+) and ind (-)). The bar depicts 100 μm in (A). ALP-positive cells were counted in ten randomly selected fields. 1 field = 12.56 mm^2^ (B). All assays were performed using three normal dermal fibroblast cell lines (CON1, CON2, and CON4) at day 4 without or with 2 μM dorsomorphin and without (open columns) or with (closed columns) osteogenic induction. Results are shown as mean ± SD. *: *P* < 0.05. These experiments were performed at least twice, and a representative data set is shown.

Dorsomorphin (also called compound C) inhibits BMP-mediated signaling and transcriptional activity, leading to blockade of BMP-induced osteoblastic differentiation [43]. Furthermore, general inhibition of BMP signaling downregulates ALP activity [25]. We examined whether dorsomorphin affected ALP activity/expression. Addition of 2 μM dorsomorphin, which is considered to be the optimum concentration for fibroblasts in the MTS assay, suppressed ALP positivity as well as ALP activity in GGCX dermal fibroblasts regardless of osteogenic induction (Fig 8A–8C). These observations suggested that calcification in GGCX involves BMP-mediated signaling, leading to ALP upregulation and osteoblastic differentiation of human dermal fibroblasts.

## Discussion

In this study, we reported a patient with a PXE-like disorder, GGCX syndrome, who had cutis laxa and sagging skin. We identified a new homozygous deletion mutation of the *GGCX* gene. The pathway leading to degeneration and mineralization of elastic fibers is not fully understood. *In vitro* study of fibroblasts derived from this patient revealed enhanced calcification accompanied by decreased gamma carboxylation of Gla proteins and upregulation of osteogenic markers. Furthermore, GGCX dermal fibroblasts exhibited accelerated calcification even without osteogenic conditions. We hypothesize that GGCX inhibits osteogenic trans-differentiation of dermal fibroblasts and prevents undesired ectopic calcification in skin.

Calcification in vessel walls possibly leads to attenuation of vessel elasticity and microcirculation insufficiency, followed by collagen accumulation in response to hypoxic conditions [44]. Alternatively, the partial osteogenic properties of GGCX dermal fibroblasts, as evidenced in this study, might involve increased collagen synthesis. Furthermore, collagen accumulation could explain the difference in cutaneous phenotypes between GGCX syndrome (i.e., cutis laxa-like sagging skin) and PXE (e.g., yellow–white papules and plaques).

Vanakker et al. [7] reported that mutations located in the exons 8, 10, and 12 which harbor the propeptide-binding site of γ-carboxylase and are important for binding of substrates, might influence the affinity of γ-carboxylase for its substrates. According to a previous report, these mutated regions of GGCX localize in the ER membrane and lumen [37]. In the current case, the homozygous c.2221delT, pS741LfsX101 mutation is localized at the C-terminus of exon 15, and this region of GGCX protein is considered to localize in the ER lumen [37]. This mutation also decreases the levels of vitamin K-dependent coagulation factors as well as mineralization in skin tissues. Taken together, this new mutation in exon 15 is suggested to be located in an important site for carboxylation similar to previous reports regarding mutations of other exons encoding proteins located in the ER lumen.

In a general pathological textbook, collagen fibers in PXE patients are split into thin fibers around denatured elastic fibers; however, there is no detailed description of collagen fibers in the dermis of a GGCX syndrome patient. In the present case of GGCX syndrome, tightly packed, thick collagen fibers accumulated around degenerative and calcified elastic fibers (Fig 2A–2F). In addition, there was calcification in blood vessel walls in the plexus of the lipo-dermal boundary, and higher magnification images revealed calcification from the internal elastic lamina to the tunica media (Fig 2H and 2I). It was reported that endothelial cells (ECs) stimulated with inflammatory cytokines produce BMP2 and endothelial microparticles with a high calcium content, leading to osteogenic transformation of vascular smooth muscle cells (VSMCs) and subsequent vascular calcification [45]. In the current case, we observed severe calcification in blood vessels in the deeper dermis (Fig 2G), and calcification seemed to be more severe around ECs than in the internal elastic lamina (Fig 2H and 2I). We did not demonstrate upregulation of BMP2 in GGCX dermal fibroblasts, and it is suggested that ECs of GGCX patients might contribute to calcification of intimal elastic lamina via BMP2 signaling. In addition, MGP is highly expressed in tube-forming ECs [46, 47], which suggests an association between calcification and intimal elastic lamina.

MMPs have an important role during the remodeling of bone and calcified tissue. Khavandgar et al. [48] reported that MGP-deficient mice have significantly increased levels of MMP-2, MMP-3, MMP-9, and MMP-13 in arteries. To determine the behavior of MMPs in the current case, we investigated the expression of MMP-2 and MMP-9 in GGCX dermal fibroblasts after osteogenic induction. Their expression levels did not change during *in vitro* ectopic calcification (S1 Fig). Diekmann et al. reported that serum MMP concentrations are elevated in patients with PXE, while MMPs are not upregulated in their fibroblasts [34]. Although we could not assess MMP expression in samples of blood, skin, or arteries from the GGCX patient, we suggest that regulation of MMPs differs among tissues or the constituent cells.

There are various reports about the roles of Gla residues in MGP. MGP may directly prevent calcification within collagen and binding of elastin fibrils to calcium or calcium crystals via its negatively charged Gla residues and phosphorylated serines residues, further protecting VSMCs from apoptosis [15]. It can also maintain VSMCs via binding to BMP2, thus playing a role in bone and cartilage development [49]. Therefore, MGP-deficient mice suffer from broken elastic fibers and ectopic mineralization [17, 30, 48]. These findings suggest that Gla residues in MGP can prevent ectopic calcification by undergoing carboxylation and directly binding to minerals, especially in the skin and cardiovascular system, in which levels of collagen and elastin are high. In the current case, accumulation of ucMGP in GGCX dermal fibroblasts was demonstrated by immunocytochemistry and an ELISA. By contrast, expression of total MGP did not differ from that in normal control fibroblasts (Fig 4). Our results imply that the level of carboxylated MGP was decreased in GGCX dermal fibroblasts considering that the ucMGP level was upregulated and the total MGP level was unchanged.

Calcification in skin and blood vessels of patients with *GGCX* gene mutations, thought to be GGCX syndrome, was suggested to be due to a lack of MGP carboxylation [15, 22, 39, 50]. In addition, Vanakker et al. [39] reported that the serum level and dermal deposition of ucMGP are increased in patients with GGCX mutations; they also demonstrated that the serum vitamin K concentration is within the normal range in such patients. By contrast, in PXE patients, the serum level of ucMGP is not upregulated [22, 39], and ucMGP appears in the mid-dermal elastorrhexis area [39]. Furthermore, these studies suggested that the serum vitamin K concentration is significantly decreased in PXE patients. In the current case, upregulation of ucMGP activity in dermal fibroblasts with mutated *GGCX* supports previous reports of an association between *GGCX* gene mutation and ucMGP. However, the behavior of ucMGP is controversial in PXE patients. The serum concentration of vitamin K, a cofactor of GGCX protein, might have a different mechanism for activation between those two diseases.

MGP, OCN, POSTN, and Gla-rich protein are Gla proteins in bone. According to the ratio of undercarboxylated /carboxylated MGP in the GGCX patient, we hypothesized that the level of carboxylated MGP was decreased. OCN mRNA was not upregulated in the GGCX patient compared with the controls according to RT-PCR (Fig 6). Other calcification-associated Gla proteins, such as OCN, POSTN, and Gla-rich protein, should have been mainly uncarboxylated and involved in disease pathogenesis because levels of the carboxylated forms of other Gla proteins, vitamin K-dependent coagulation factors (factors II, VII, IX, and X), were obviously decreased in the patient’s blood. This suggested that the patient’s mutation similarly inactivated the physiological function of these bone-associated Gla proteins.

Regardless of osteogenic induction, GGCX dermal fibroblasts showed enhanced calcification with increased mRNA expression of ALP, BMP6, RUNX2, POSTN, and COL10A1 (Figs 5 and 6). mRNA expression levels of OSX and COL1A2 in GGCX dermal fibroblasts were less than the mean + 2SD of those in normal dermal fibroblasts without osteogenic induction. However, after osteogenic induction, expression of OSX and COL1A2 increased by 3.9- and 2.9-fold, respectively (Fig 6). RUNX2 and OSX are transcription factors necessary for osteoblast differentiation and chondrocyte maturation [26]. COL1A2 and ALP are expressed in the early stage of osteoblast differentiation, whereas OCN is highly expressed in mature osteoblasts and during osteocyte differentiation [29]. However, OCN was not expressed in GGCX dermal fibroblasts even with osteogenic induction. Taken together, GGCX dermal fibroblasts may display properties of osteogenic mesenchymal cells, including osteoprogenitor cells, preosteoblasts, and osteoblasts, but not osteocytes.

ALP expression was increased in GGCX dermal fibroblasts at 4 days with or without osteogenic induction, but not in PXE dermal fibroblasts (Fig 7A and 7B). On the other hand, Boraldi et al. [22] reported that ALP expression is increased in PXE fibroblasts even without osteogenic induction. We considered three reasons for this difference. First, the difference may be due to the culture conditions. Dabisch-Ruthe et al. [23] reported that a low concentration of inorganic pyrophosphate (PPi) in culture medium is suitable for calcification accompanied by elevation of ALP in PXE fibroblasts. The concentrations of PPi in the current study and in the report by Boraldi et al. are uncertain. Second, the time point of ALP evaluation differed. ALP activity was measured at 48 hours after cells reached confluency in the report by Boraldi et al., but at 96 hours after cell seeding in the current study. Birmingham et al. [42] reported that ALP activity peaks at 2 or 3 days regardless of treatment. It is possible that ALP activity increased at 48 hours after PXE dermal fibroblasts reached confluency in the current study. Third, the characteristics of the PXE patients from whom fibroblasts were derived differed. Boraldi et al. studied six Italian PXE patients, although details of their gene mutations were not reported. Gheduzzi et al. [51] reported that the large majority of 54 Italian PXE patients harbored *ABCC6* mutations located between exons 24 and 30. We studied Japanese PXE patients with mutations located in exons 5, 9, and 19; one case had a homozygous stop mutation, and the other two cases had compound heterozygous mutations (S2 Table). Unfortunately, we could not determine whether the degree of ALP expression differed according to the location of the mutation.

These three reasons may explain why ALP expression and activity were not elevated in PXE dermal fibroblasts without osteogenic induction (Fig 7), opposite to the finding of Boraldi et al. Calcification seems to depend on many factors in PXE patients, including ALP expression. Our results also suggest that calcification occurs more readily in GGCX dermal fibroblasts than in PXE dermal fibroblasts via ALP signaling in our experimental settings, supporting the mesenchymal and osteogenic trans-differentiation of GGCX fibroblasts.

In this study, we had a limited sample of GGCX dermal fibroblasts; therefore, the pathway by which loss of GGCX carboxylase activity leads to degeneration and calcification of elastic fibers in GGCX syndrome was not clarified. Nevertheless, this study revealed the increased osteogenic differentiation of GGCX dermal fibroblasts. Taken together, it is intriguing to explore the mechanisms by which substrates of GGCX may prevent trans-differentiation into osteogenic cells as well as ectopic calcification.

## Supporting information

S1 Fig mRNA levels of MMP-2 and MMP-9. RT-PCR analysis indicated that the mRNA level of MMP-2 in GGCX dermal fibroblasts (Pt) was similar to that in normal dermal fibroblasts (CON). The mRNA level of MMP-9 in CON was significantly higher with induction than without induction. Furthermore, there was almost no signal in Pt. *MMP-9/GAPDH* ratios are shown as means ± SD (n = 3).
(TIF)

S2 Fig Predicted sequence of the mutated GGCX protein. The mutated GGCX protein has 100 abnormal amino acids at the C-terminus (letters in red), resulting in the addition of an extra 82 amino acids at the C-terminus.
(TIF)

S3 Fig mRNA level of mutated GGCX. RT-PCR analysis indicated that the mRNA level of mutated GGCX in GGCX dermal fibroblasts (Pt) was unchanged. *GGCX/GAPDH* ratios are shown as means ± SD (n = 3).
(TIF)

S1 Table Primer sequences for the *GGCX* gene. (TIF)

S2 Table Profiles of PXE patients. The three PXE patients had typical skin symptoms, degeneration and calcification of elastic fibers in the dermis, and angioid streaks. All three PXE patients had two nonsense mutations, leading to loss of function of the ABCC6 molecule.
(TIF)
