# Supplementary figures and images for: Calcification in dermal fibroblasts from a patient with GGCX syndrome accompanied by upregulation of osteogenic molecules
Source: PLoS One. 2017 May 11;12(5):e0177375. doi: 10.1371/journal.pone.0177375 (PMC5426700; doi:10.1371/journal.pone.0177375)

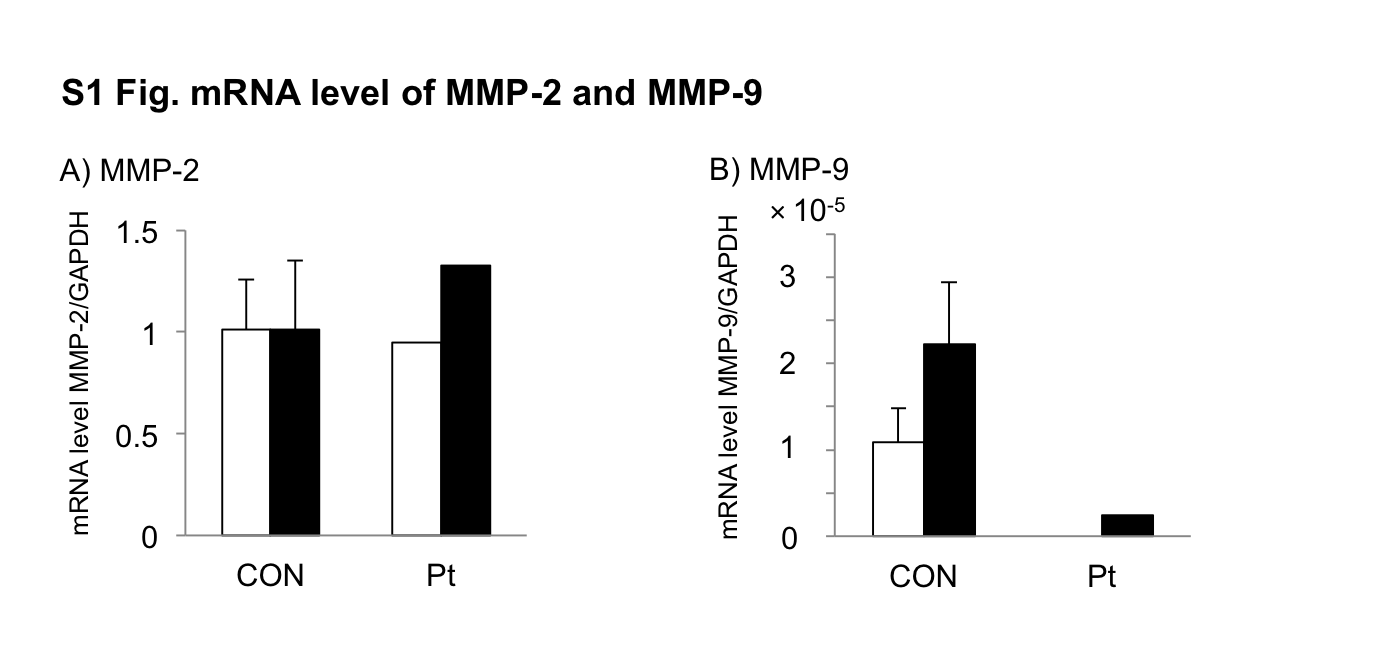

Supplement: S1 Fig — RT-PCR analysis indicated that the mRNA level of MMP-2 in GGCX dermal fibroblasts (Pt) was similar to that in normal dermal fibroblasts (CON). The mRNA level of MMP-9 in CON was significantly higher with induction than without induction. Furthermore, there was almost no signal in Pt. MMP-9/GAPDH ratios are shown as means ± SD (n = 3). (TIF) [file pone.0177375.s001.tif]

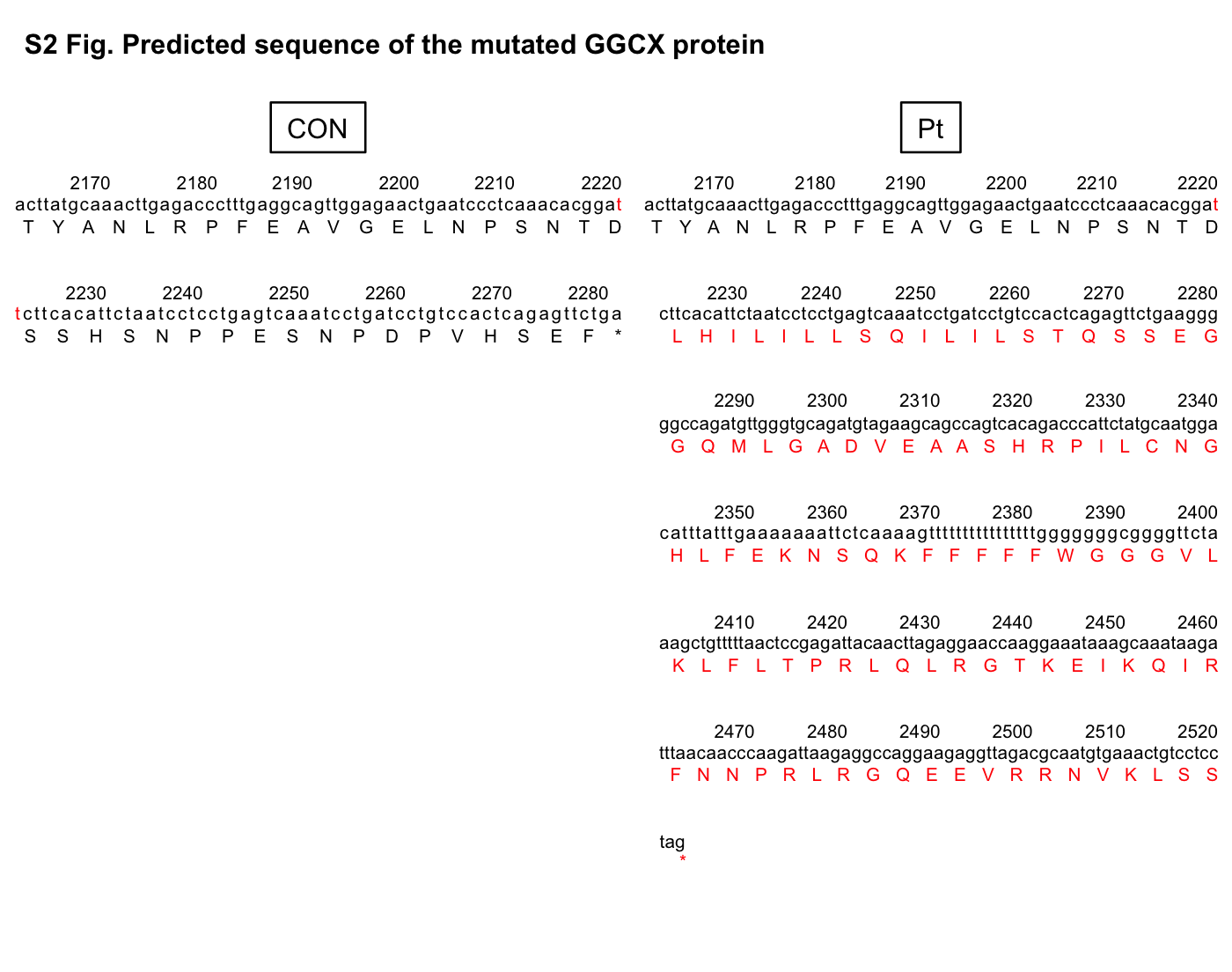

Supplement: S2 Fig — The mutated GGCX protein has 100 abnormal amino acids at the C-terminus (letters in red), resulting in the addition of an extra 82 amino acids at the C-terminus. (TIF) [file pone.0177375.s002.tif]

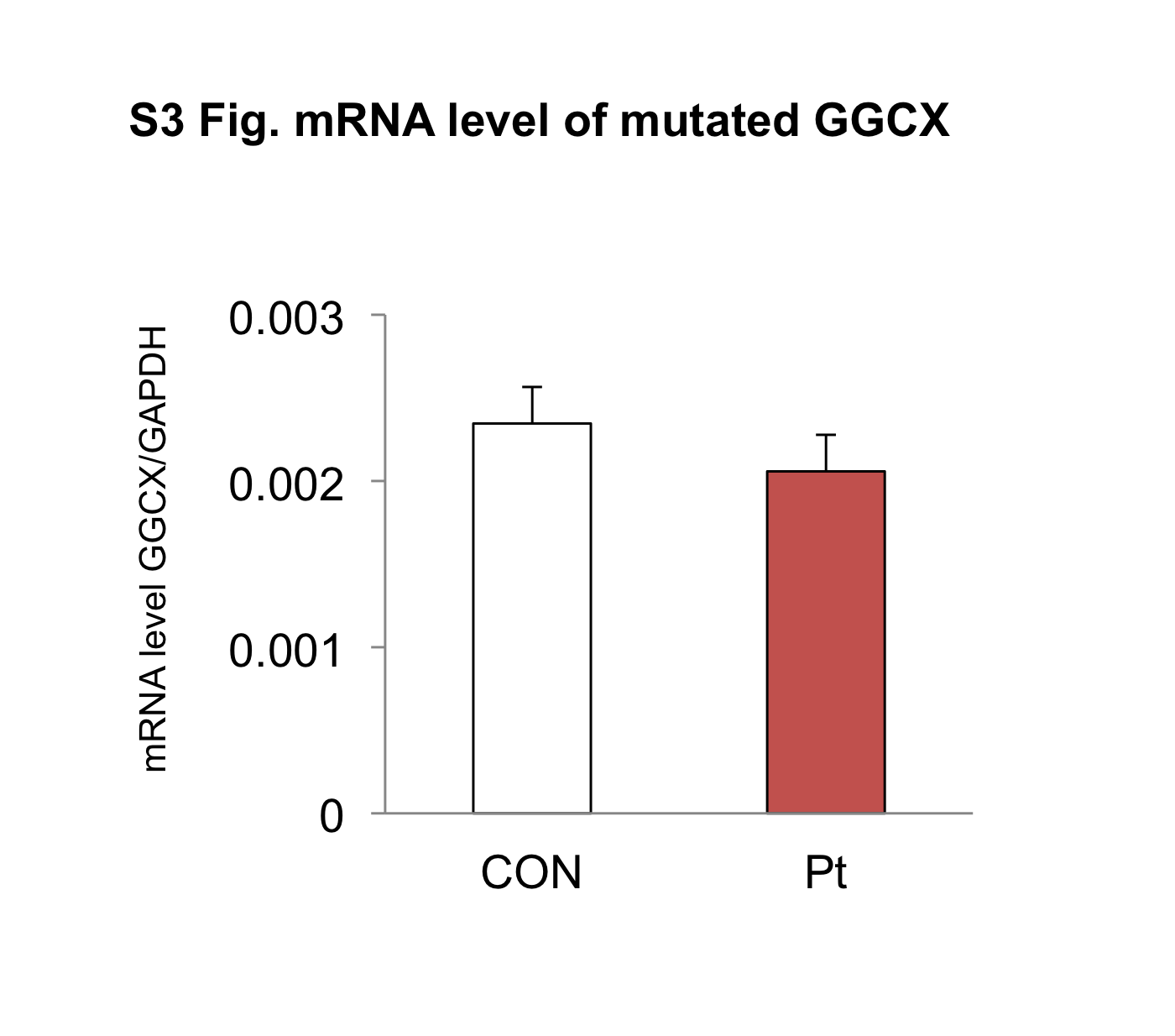

Supplement: S3 Fig — RT-PCR analysis indicated that the mRNA level of mutated GGCX in GGCX dermal fibroblasts (Pt) was unchanged. GGCX/GAPDH ratios are shown as means ± SD (n = 3). (TIF) [file pone.0177375.s003.tif]

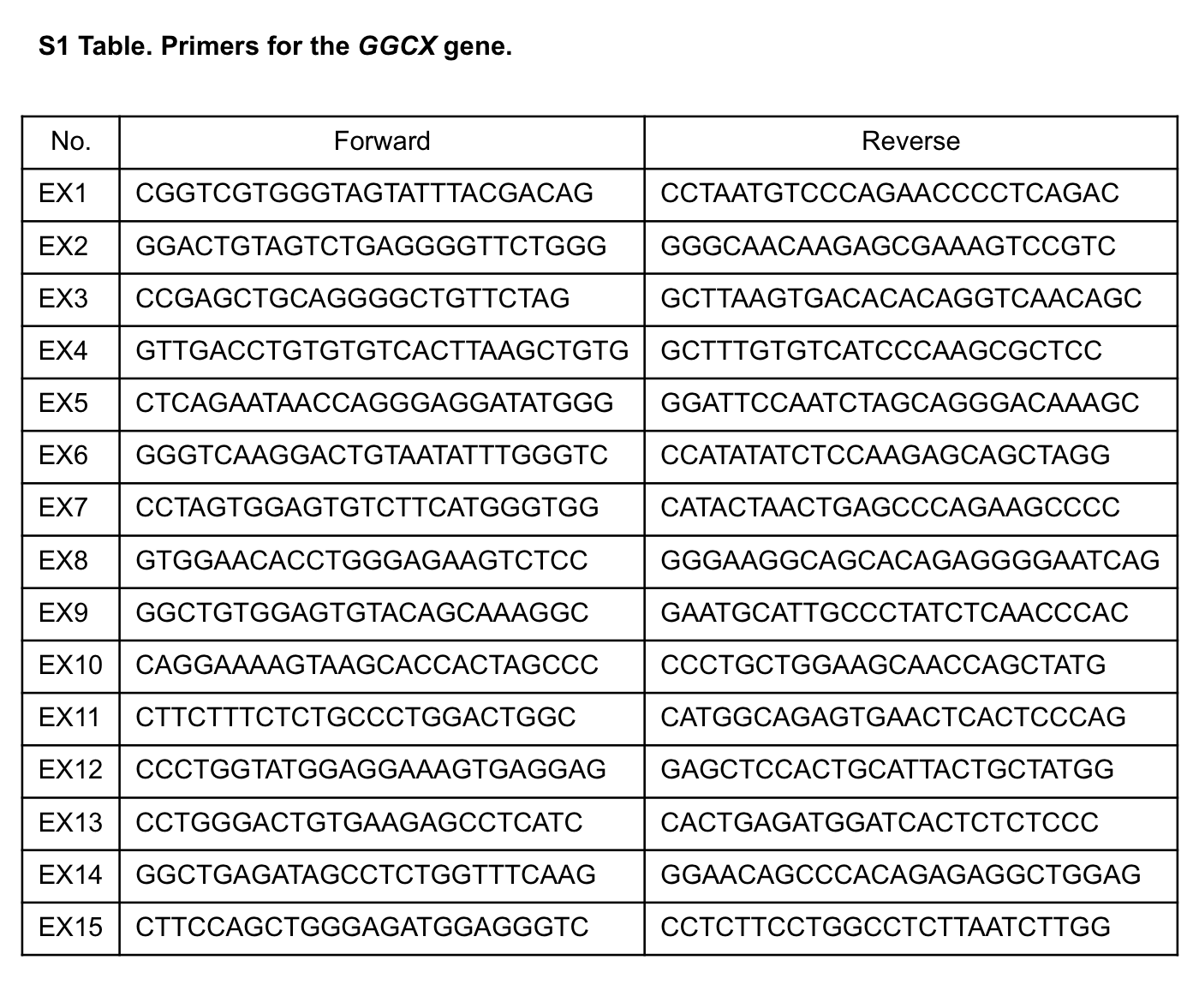

Supplement: S1 Table — (TIF) [file pone.0177375.s004.tif]

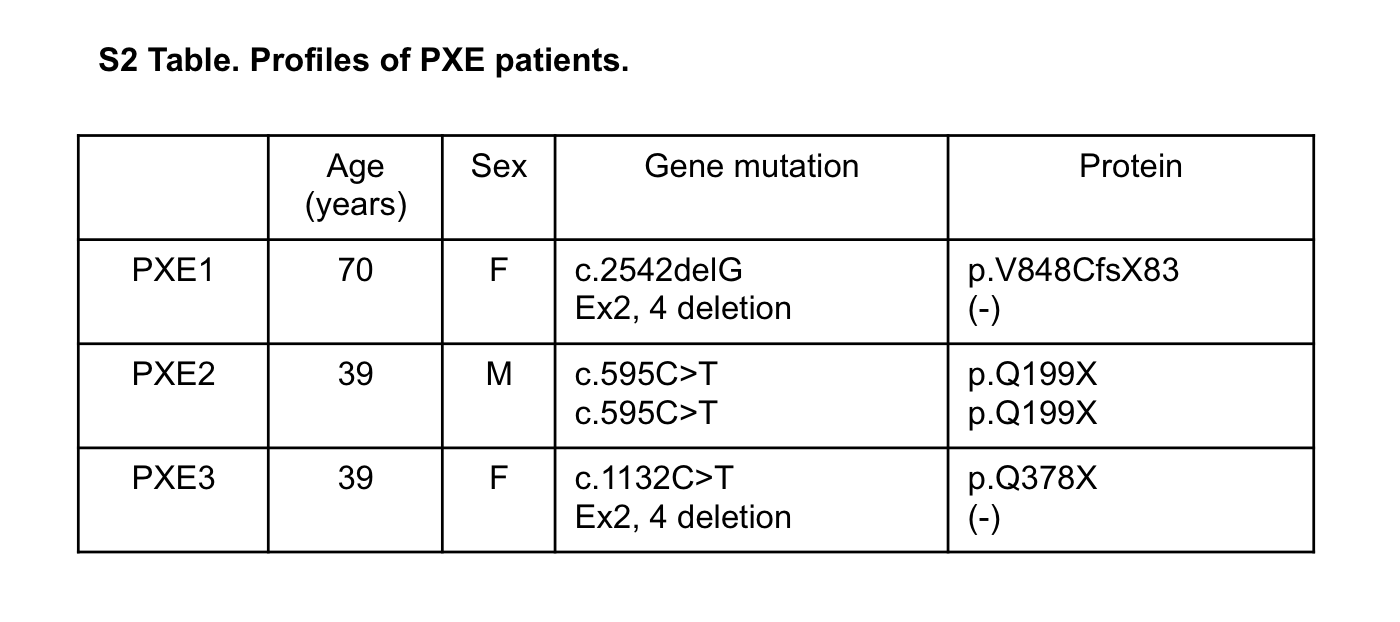

Supplement: S2 Table — The three PXE patients had typical skin symptoms, degeneration and calcification of elastic fibers in the dermis, and angioid streaks. All three PXE patients had two nonsense mutations, leading to loss of function of the ABCC6 molecule. (TIF) [file pone.0177375.s005.tif]
